# Effects of organic extracts of six Bangladeshi plants on *in vitro* thrombolysis and cytotoxicity

**DOI:** 10.1186/1472-6882-13-25

**Published:** 2013-01-30

**Authors:** M Atiar Rahman, Rabeya Sultana, Talha Bin Emran, M Saiful Islam, M Ashiqur Rahman, Joti Sankhar Chakma, Harun-ur Rashid, Chowdhury Mohammad Monirul Hasan

**Affiliations:** 1 Department of Biochemistry and Molecular Biology, University of Chittagong, Chittagong, 4331, Bangladesh

**Keywords:** Thrombolysis, Clausena suffruticosa, Leea indica, Leucas aspera, Streptokinase

## Abstract

**Background:**

Thrombus formed in blood vessels lead to atherothrombotic diseases such as myocardial or cerebral infarction. Thrombolytic agents are used to dissolve the already formed clots in the blood vessels; however, these drugs sometimes cause serious and fatal consequences. Herbal preparations have been used since ancient times for the treatment of several diseases although they show little toxicity in some cases. Aqueous extracts of herbs used in thrombolysis have been reported before with cytotoxic data, however, the organic extracts of herbs have not been documented. This study aims to investigate whether organic extracts possess thrombolytic properties with minimal or no toxicity.

**Methods:**

An *in vitro* thrombolytic model was used to check the clot lysis effect of six Bangladeshi herbal extracts viz., *Ageratum conyzoides* L., *Clausena suffruticosa*, *Leea indica* (Burm.f.) Merr., *Leucas aspera* Willd., *Senna sophera* L. Roxb., and *Solanum torvum* Swartz. using streptokinase as a positive control and water as a negative control. Briefly, venous blood drawn from twenty healthy volunteers was allowed to form clots which were weighed and treated with the test plant materials to disrupt the clots. Weight of clot after and before treatment provided a percentage of clot lysis. Cytotoxicity was screened by brine shrimp lethality bioassay using vincristine sulfate as positive control.

**Results:**

Using an *in vitro* thrombolytic model, *Ageratum conyzoides, Clausena suffruticosa, Leea indica, Leucas aspera, Senna sophera* and *Solanum torvum* showed 18.12 ± 2.34%, 48.9 ± 2.44%, 39.30 ± 0.96%, 37.32 ± 2.00%, 31.61 ± 2.97% and 31.51 ± 0.57% and clot lysis respectively. Among the herbs studied *Clausena suffruticosa, Leea indica* and *Leucas aspera* showed very significant (p < 0.0001) percentage (%) of clot lysis compared to reference drug streptokinase (75.00 ± 3.04%). In brine shrimp cytotoxic assay, the extracts *Ageratum conyzoides, Clausena suffruticosa, Leea indica, Leucas aspera, Senna sophera* and *Solanum torvum* showed LC_50_ values 508.86 ± 6.62,41.16 ± 1.26, 2.65 ± 0.16, 181.67 ± 1.65, 233.37 ± 7.74 and 478.40 ± 3.23 μg/ml, respectively, with reference to vincristine sulfate (LC_50_ 0.76 ± 0.04).

**Conclusion:**

Through our study it was found that *Clausena suffruticosa*, *Leea indica* and *Leucas aspera* possessed effective thrombolytic properties whereas *Senna sophera* and *Solanum torvum* showed moderate to mild thrombolytic effects while *Ageratum conyzoides* showed no significant effect. No extract was found cytoxic compared to positive control. *Clausena suffruticosa*, *Leea indica* and *Leucas aspera* could be incorporated as a thrombolytic agent with *in vivo* effects to improve the atherothrombotic patients. However, *Clausena suffruticosa* could be the best one to use in this purpose.

## Background

Coronary artery thrombosis has been treated by urokinase (UK), streptokinase (SK) or tissue plasminogen activators (t-PA) which are widely used clinical thrombolytic agent for the treatment of severe or massive deep venous thrombosis, pulmonary embolism, myocardial infarction, and occluded intravenous or dialysis cannulas [1]. Although UK and SK are widely used in India, Bangladesh and other developing countries due to lower cost [2] as compared to other thrombolytic drugs but, the use is associated with high risk of bleeding intracranial hemorrhage, severe anaphylactic reaction and lacks specificity [3]. Moreover, these drugs are not used in patients who have undergone surgery or those with a history of nervous lesions, gastrointestinal bleeding or hypertension [3]. However, as a result of immunogenicity multiple treatments with SK in a given patient are restricted [4]. Thrombolytic therapy with recombinant t-PA is effective in acute myocardial infarction, but the treatment is limited by a fairly slow reperfusion rate and frequent early reocclusions. Moreover, the platelet-rich thrombi are highly resistant to lysis by t-PA [5]. Another thrombolytic agent fucoidan, a branched sulfated fucan extracted from brown seaweeds with anticoagulant and antithrombotic effects mediated by direct thrombin inhibition has been reported recently [1].

However, herbal drugs are wide-spoken as green medicine for their safe and dependable health care paradigms. The traditional herbal medicines increased an uprising interest since couple of decades due to their incredible pharmacological activities, economic viability and less side effects in different healthcare management system [6]. Thus, tremendous efforts have also been directed towards the discovery and development of natural products with antiplatelet [7,8], anticoagulant [9,10], antithrombotic [11] and thrombolytic activity of the plants not documented. Epidemiologic studies have provided evidence that foods with experimentally proved antithrombotic effect could reduce risk of thrombosis. Some plants or plant parts showing thrombolytic activity have also been reported [12,13]. *Ageratum conyzoides* L. (Goat weed or white weed or Chuva), *Clausena suffruticosa* (Kalomoricha)*, Leea indica* (Burm.f.) Merr. (Achilagach or Arengi), *Leucas aspera* Willd. (Darkolos or Dandokolos), *Senna sophera* L. Roxb. (Kanduak or Khuksu), and *Solanum torvum* Swartz. (Titbegun) are native to Bangladesh. They are used as traditional medicines for cardiac diseases and blood purification. Recently, *A. conyzoides* was reported to have hematological and lymphocyte increasing activity [14]. Roots, leaves and seeds of *C. suffruticosa* are used in the treatment of bleeding and cerebrospinal meningitis [15,16]. Flowers, roots and leaves of *L. indica* are used as anti-inflammatory, anxiolytic and cardioprotective [17-19]. *Leucas aspera* stem and whole plant were found to possess anti-inflammatory and blood purifying effects [20,21]. *Solanum torvum* aqueous extract is reported to contribute in increasing red blood cells and hemoglobin concentration above baseline values within 24 days [22]. But their thrombolytic effects and usability in context of toxicity have not been studied at all.

This study aims to investigate the ethanol extracts of the aforementioned six Bangladeshi medicinal plants viz., *Ageratum conyzoides* (*A. conyzoides*), *Clausena suffruticosa* (*C. suffruticosa*)*, Leea indica* (*L*. *indica*)*, Leucas aspera* (*L. aspera*), *Senna sophera* (*S. sophera*) and *Solanum torvum* (*S. torvum*) for their clot lysis (thrombolytic activity) and cytotoxic properties by using *in vitro* models.

## Methods

### Plant collection and identification

Whole plants of *A. conyzoides* (Accession No. 36073), root of *C. suffruticosa* (Accession No. 32909), leaf of *L. indica* (Accession No. 36078), whole plants of *L. aspera* (Accession No. 36070), leaf of *S. sophera* (Accession No. 36072) and fruits of *S. torvum* (Accession No. 36071) were collected from different parts of Chittagong region, Bangladesh. The plants were identified by Dr. Shaikh Bokhtear Uddin, Taxonomist and Associate Professor, Department of Botany, University of Chittagong. The sample specimens of the identified plants have been preserved in the national herbarium with the mentioned accession numbers.

### Chemicals and reagents

To the commercially available lyophilized Streptokinase (SK) vial (Durakinase, Dongkook Phama. Co. Ltd, South Korea) of 15 00000 I.U., 5 ml sterile distilled water was added and mixed properly. This suspension was used as a stock from which 100 μl (30,000 I.U) was used for *in vitro* thrombolysis. Absolute ethanol (99.50%) and vincristine sulfate (VS) were purchased from Sigma-Aldrich, Munich, Germany.

### Extract preparation

Each of the plant materials was dried and ground (Moulinex Blender AK-241, Moulinex, France) into powder (40-80mesh, 500 g) and soaked for 7 days with 2–3 days interval in 2.0 L of ethanol at room temperature (23 ± 0.5°C). Filtrate obtained through cheesecloth and Whatman filter paper No. 1 was concentrated under reduced pressure at the temperature below 50°C using rotary evaporator (RE 200, Sterling, UK). The extracts (yield 4.4–5.6% W/W) were all placed in glass Petri dishes (90 X 15 mm, Pyrex, Germany). A 100 mg each of the extracts was suspended in 10 ml distilled water and the suspension was shaken vigorously on a vortex mixer. The suspension was kept overnight and decanted to remove the soluble supernatant, which was filtered through a 0.22-μm syringe filter. A 100 μl of this aqueous preparation was added to the microcentrifuge tubes containing the clots to check thrombolytic activity. The same concentration (10 mg/ml) of extracts was prepared for screening the cytotoxic properties.

### Blood specimen

Whole blood (4 ml) was drawn from healthy human volunteers (*n* = 20) without a history of oral contraceptive or anticoagulant therapy using a protocol approved by the Institutional Ethics Committee of Chittagong University, faculty of medicine. An earlier consent, approval number HET-CU2011/1, was taken from the faculty of medicine, University of Chittagong, for collection of blood samples from Human volunteers. Blood collection and preservation were conducted by Dr. M Rafiqur Rahman (Pathologist, faculty of Medicine, University of Chittagong). A 500 μl of blood was transferred to each of the eight previously weighed microcentrifuge tubes to form clots.

### Statement on informed consent of the donors

The volunteer donors were supplied a consent form which informed the title of the research project, name and detail contact of investigators as well as purpose of the research. Description of the research mentioning step-by-step brief of the proposed research, inclusion and exclusion criteria of the donors, whether donors will receive any therapy or not, volume of blood to be taken, possible discomfort of the puncture sites, time required for the blood sampling. Explanation was made on if future use of the research data beyond the current study is anticipated, whether this is a focus group if so the Principal Investigator should put a procedure in place in which the researchers caution people about the limit on confidentiality. Access to Research Information regarding who would have access to the collected sample, information regarding retention of sample and schedules for their disposal were also detailed. It was indicated to the consent form that the volunteers might refuse to donate blood at any time. Donor whether could withdraw his sample data was disclosed. The sample was restricted for that individual study not for future research projects was presented in the consent form. Potential harm, injuries, discomforts or inconvenience associated with donors in this study was added as informed consent statement. If there was known harm to the donors, the potential harm, current knowledge regarding the probability of the occurrence of the harm, clinical importance of the harm; and any relevant knowledge regarding the probability of reversibility; for example the possibility of bruising or swelling while giving blood, or some other discomforts at the site where blood is drawn and that there might be minimal chance of infection, and that these discomforts were brief and transient were also added. Potential benefits of the donors, not directly, but the society in general or individuals with a similar condition might benefit from the results of this study was explained. Treatment alternative and possibility of the research was described. Confidentiality statement was included in the consent form in the way that “confidentiality will be respected and no information that discloses the identity of the participant will be released or published without consent unless required by law of states. The legal obligation includes a number of circumstances, such as suspected child abuse and infectious disease, expression of suicidal ideas where research documents are ordered to be produced by a court of law and where researchers are obliged to report to the appropriate authorities. In those rare instances where it will not be possible to assure complete confidentiality”, the limits on this obligation were carefully explained. Reimbursement issue was also mentioned whether the donors or their parents may be offered money for reasonable out-of-pocket expenses for example, transportation costs, meals, etc. Finally detail contact (Name, area code and phone number) of investigators was provided in case of any questions of the donors about this study. The consent form was concluded with major questions on above disclosures in Yes/NO form followed by the signature (with date) of the donor.

### Clot lysis

Experiments for clot lysis were carried as reported earlier [23]. Briefly, four ml venous blood drawn from the healthy volunteers was distributed in eight different pre weighed sterile microcentrifuge tube (0.5 ml/tube) and incubated at 37°C for 45 min. After clot formation, serum was completely removed without disturbing the clot and each tube having clot was again weighed to determine the clot weight (clot weight = weight of clot containing tube –weight of tube alone). To each microcentrifuge tube containing pre-weighed clot, 100 μl of organic extracts of the six plants (*A. conyzoides, C. suffruticosa, L. indica, L. aspera, S. sophera* and *S. torvum*) were added separately. As a positive control, 100 μl of SK and as a negative non-thrombolytic control, 100 μl of distilled water were separately added to the control tubes numbered. All the tubes were then incubated at 37°C for 90 min and observed for clot lysis. After incubation, fluid released was removed and tubes were again weighed to observe the difference in weight after clot disruption. Difference obtained in weight taken before and after clot lysis was expressed as percentage of clot lysis. The experiment was repeated with the blood samples of the 20 volunteers.

### Cytotoxicity assay

Brine shrimp bioassay was carried out with the method as described by Meyer et al. (1982) [24] to investigate the cytotoxicity of the extracts. The dried extract preparations were re-dissolved in DMSO to obtain a solution of 10 mg/ml which was subjected to serial dilution to get the concentrations between 20 μg/ml- 800 μg/ml. A 5.0 ml of artificial sea water was added into all the test tubes. Simple zoological organism (*Artemia salina*) was used as a convenient monitor for cytotoxic screening. The eggs of the brine shrimps were collected from the Institute of Marine Science and Fisheries, University of Chittagong, Bangladesh and hatched in artificial seawater (prepared by using sea salt 38 g/L and adjusted to pH 8.5 using 1N NaOH) under constant aeration for 24 h under the light. The hatched shrimps were allowed to grow by 48 h to get shrimp larvae called nauplii. After 48 h, active nauplii were attracted to one side in a glass petri dish by using a micropipette. The nauplii were then separated from the eggs by aliquoting them in another glass petri dish containing artificial sea water and used for the assay. Suspension containing 20 nauplii was added into each test tube and was incubated at room temperature (25±1°C) for 12 h under the light. The tubes were then examined after 24 h and the number of surviving larvae in each tube was counted with the aid of a 3X magnifying glass. Experiments were conducted along with VS in a set of three tubes per dose. The concentration that would kill 50% of the nauplii (LC_50_) was determined from a linear regression equation using the software “BioStat-2009”.

### Statistical analysis

The significance between % clot lysis by SK and plant extracts, LC_50_ values by VS and extracts was tested by the paired t-test analysis using the software SPSS, version 19.0 (SPSS for Windows, Version 18.0, IBM Corporation, New York, USA). Data are expressed as mean ± standard deviation. The mean difference between positive and negative control was considered significant at p < 0.05.

## Results

**Figure 1:**
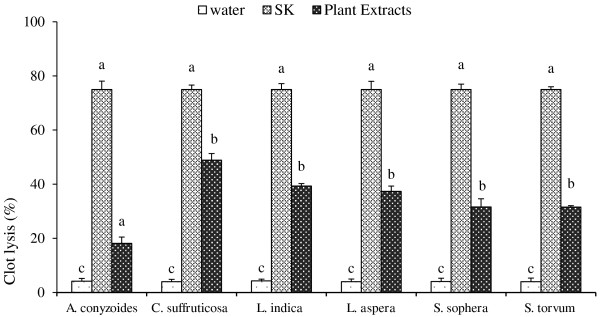
**Clot lysis by streptokinase, water and various organic extracts.** Effects of drugs on dissolution of clots prepared from blood of normal individuals. Maximum clot lysis (75.00 ± 3.04%) was observed in clot treated with streptokinase (SK). Among herbal drugs *C. suffruticosa* (Kalomoricha), *L. indica* (Achilagach) and *L. aspera* showed 48.90 ± 2.44%, 39.30 ± 0.96% and 37.32 ± 2.00% clot lysis, respectively. Water (as a negative control) showed 4.19 ± 0.37% clot lysis. Data were processed with *Tukey’s post hoc test* for multiple comparisons, SPSS for windows, version 18.0, p < 0.05) from each other.

Addition of 100 μl SK (positive control) to the clots along with 90 min of incubation at 37°C, showed 75.00 ± 3.04% clot lysis. However, distilled water (negative control) treated-clots showed only negligible clot lysis (4.19 ± 0.37%). The mean difference in clot lysis percentage between positive and negative control was very significant (p value < 0.0001). Treatment of clots with *A. conyzoides, C. suffruticosa*, *L. indica*, *L. aspera*, *S. sophera and S. torvum* extracts provided the clot lysis 18.12 ± 2.34%, 48.90 ± 2.44%, 39.30 ± 0.96%, 37.32 ± 2.00%, 31.61 ± 2.97% and 31.51 ± 0.57%, respectively. The mean percentage of clot lysis by *C. suffruticosa*, *L. indica and L. aspera* was statistically very significant (p value < 0.0001) compared to those of both positive control SK and negative control water. *Senna sophera* and *S. torvum* showed relatively lower percentage of clot lysis although the values were significant (p value < 0.001) compared to those of both positive control SK and negative control water. However, *A. conyzoides* did not show any significant clot lysis effect. Percent clot lysis obtained after treating the clots with different organic extracts and appropriate controls is shown in Figure 1.

**Figure 2:**
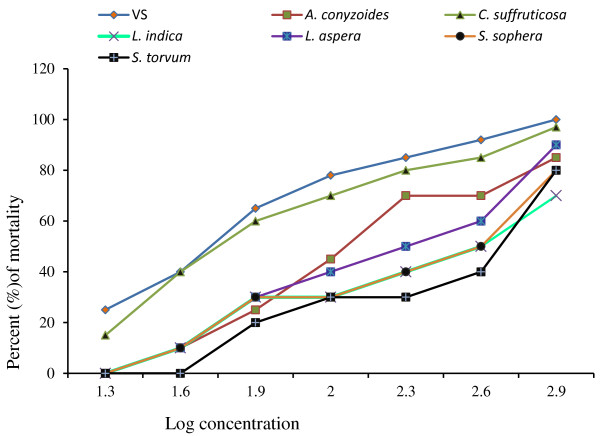
**Brine shrimp mortality by VS and different organic extracts.** Percent mortality of brine shrimps of six plant extracts and standard cytotoxic agent vincristine sulfate (VS). Data are shown as mean ± SD of twenty shrimps for each concentration. Mortality achieved by the extracts of *A. conyzoides, C. suffruticosa, L. indica, L. aspera, S. sophera* and *S. torvum* are lower than that by VS. Data were processed with *Tukey’s post hoc test* for multiple comparisons, SPSS for windows, version 18.0, p < 0.05) from each other.

**Figure 3:**
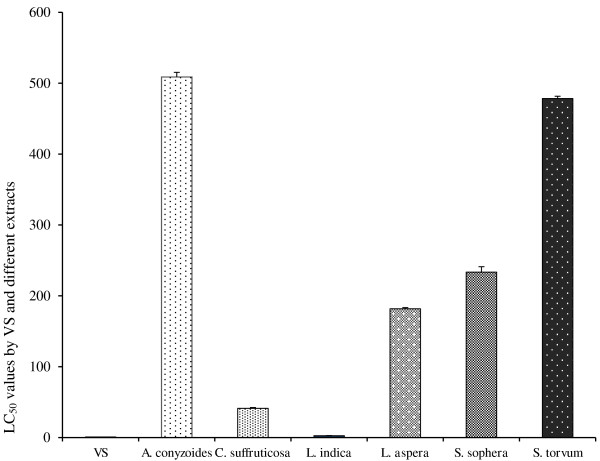
**Brine shrimp lethality by VS and different organic extracts.** Lethal concentration (LC_50_) of VS and different organic extracts for brine shrimp nauplii (*Artemia salina*). Data are shown as mean ± SD of twenty shrimps for each concentration. *A. conyzoides, C. suffruticosa, L. indica, L. aspera, S. sophera* and *S. torvum* showed LC_50_ values 508.86 ± 6.62, 41.16 ± 1.26, 2.65 ± 0.16, 181.67 ± 1.65, 233.37 ± 7.74 and 478.40 ± 3.23 μg/ml, respectively, with reference to vincristine sulfate (LC_50,_ 0.76 ± 0.04. Data were processed with *Tukey’s post hoc test* for multiple comparisons, SPSS for windows, version 18.0, p < 0.05) from each other.

The regression analysis for brine shrimp bioassay was presented in Table 1. Comparative mortality of brine shrimps and LC_50_values for different extracts in reference to control (VS) was shown in Figures 2 and 3, respectively. No extract was found to be significantly toxic compared to positive control. The *L. indica* extract had a smallest LC_50_ value of 2.65 ± 0.16 μg/ml which was significantly (p < 0.01) different from that (0.76 ± 0.04 μg/ml) of positive control indicating that the extract is not toxic (Figure 2).

**Table 1:** Calculation of LC_50_ values, confidence limits, regression equations and chi square values for different extracts with reference to vincristine sulfate

**Sample**	**LC_50_ (μg/ml)**	**Range of confidence limit (μg/ml)**	**Regression equation**	**Chi square**
VS	0.76 ± 0.04	0.57–0.82	Y= 3.16 + 2.98X	0.63
*A. conyzoides*	508.86 ± 6.62	390.12–655.23	Y = − 2.16 + 2.65* X	0.70
*C. suffruticosa*	41.16 ± 1.26	31.66–52.33	Y = 2.92 + 4.03*X	1.58
*L. indica*	2.65 ± 0.16	2.25–2.69	Y = 45.98*X + S32	1.70
*L. aspera*	181.67 ± 1.65	125.12–265.96	Y = 56.28X - 76.73	0.76
*S. sophera*	233.37 ± 7.74	156.74–361.99	Y = 51.49X - 71.00	1.15
*S. torvum*	478.40 ± 3.23	345.78–611.01	Y = 52.09* X - 79.45	0.93

## Discussion

Plant-derived medicines have a long history of use for the prevention and treatment of human diseases. Today, many pharmaceuticals currently approved by the Food and Drug Administration (FDA) have origins to plant sources. A major role for plant-derived compounds based on the reported immunomodulatory effects has emerged in recent times and has led to the rigorous scientific examination to determine efficacy and safety [25]. A number of plants source especially several fruits and vegetables have been studied for their supplements having anticoagulant, antiplatelet and fibrinolytic activity and there is evidence that consuming such food leads to prevention of coronary events and stroke [26-29]. Some of these plant products are modified further with recombinant technology [30] to make them more effective and site specific.

In our thrombolytic assay, the comparison of positive control with negative control clearly demonstrated that clot dissolution does not occur when water was added to the clot. When compared with the clot lysis percentage obtained through SK and water, an extremely significant (p value < 0.0001) thrombolytic activity was observed after treating the clots with *C. suffruticosa, L. indica* and *L. aspera* extracts. However, the clot lysis values for *L. indica* and *L. aspera* were lower than that of *C. suffruticosa*. It is evidenced that there are bacterial contaminants of plants which have plasminogen receptors that bind plasminogen. Cell surface bound plasminogen is easily activated to plasmin, which could lead to fibrinolysis [31]. Bacterial plasminogen activator: staphylokinase, streptokinase, act as cofactor molecules that contribute to exosite formation and enhance the substrate presentation to the enzyme. Staphylokinase activates plasminogen to dissolve clots, also destroys the extracellular matrix and fibrin fibers that hold cells together [32-34]. Interestingly the *C. suffruticosa, L. indica* and *L. aspera* are known to have antibacterial activity which is conducted against ten bacterial strains including *E. coli* and *Staphylococcus aureus*[15,35,36]. Thus, the observed thrombolytic effects could be linked to the antibacterial activity of the plants. In context of the above, it would be interesting to investigate the mechanism underlying the clot lytic effects demonstrated by *C. suffruticosa, L. indica* and *L. aspera* extract.

Toxicity of plant materials is a major concern to scientists and medical practitioners [37-39] and therefore cytotoxic assay was conducted in this study to determine the toxicity profile of the plant extracts through the Brine Shrimp Lethality (LC_50_, 24 h) test. Lagarto [40] demonstrated a good correlation (r = 0.85; *P* < 0.05) between the LC_50_ of the brine shrimp lethality test and the acute oral toxicity assay in mice. Based on that correlation, brine shrimp lethality LC_50_ < 10 μg/ml (LD_50_ between 100 and 1000 mg/kg) is considered as the cutoff value of cytotoxicity [6,40]. According to the measured LC_50_ values of the extracts no one was found severely lethal or toxic to be processed as pharmaceutical products in thrombolytic uses. However, the extremely significant effect of *C. suffruticosa* demonstrates it to be the best thrombolytic component for further processing.

## Conclusion

In conclusion, on the basis of beneficial effects of *C. suffruticosa, L. indica* and *L. aspera* in the Bangladeshi traditional medicine which was validated in this study, these plant extracts possessed great blood clots lytic activity *in vitro*; however, *in vivo* clot dissolving property and active component(s) of *C. suffruticosa* for clot lysis are yet to be investigated. Further work will establish whether or not, phytochemicals derived from this plant could be incorporated as a thrombolytic agent for the improvement of the patients suffering from atherothrombotic diseases.

## Competing interests

The authors declare that they have no competing interests.

## Authors’ contributions

MAR carried out the study design, data collection, data interpretation, manuscript preparation, statistical analysis and research grant collection. RS, JSC, TBI, MAsR, MSI, MT and HUR participated in experiments, data collection, literature search and manuscript preparation. CMMH has provided assistance in taxonomical identification and collections of voucher specimen’s numbers for all the plants. CMMH also supervised the study design, data interpretation and literature search. All authors read and approved the final version of the manuscript.

## Pre-publication history

The pre-publication history for this paper can be accessed here:

http://www.biomedcentral.com/1472-6882/13/25/prepub
